# RFX transcription factors control a miR-150/PDAP1 axis that restrains the proliferation of human T cells

**DOI:** 10.1371/journal.pbio.3001538

**Published:** 2022-02-10

**Authors:** Michele Chirichella, Niccolò Bianchi, Emina Džafo, Elena Foli, Francesco Gualdrini, Amy Kenyon, Gioacchino Natoli, Silvia Monticelli

**Affiliations:** 1 Institute for Research in Biomedicine (IRB), Università della Svizzera italiana (USI), Bellinzona, Switzerland; 2 Graduate School for Cellular and Biomedical Sciences, University of Bern, Bern, Switzerland; 3 IEO, European Institute of Oncology IRCCS, Department of Experimental Oncology, Milan, Italy; 4 Humanitas University, Milan, Italy; Children’s Hospital of Philadelphia and The University of Pennsylvania School of Medicine, UNITED STATES

## Abstract

Within the immune system, microRNAs (miRNAs) exert key regulatory functions. However, what are the mRNA targets regulated by miRNAs and how miRNAs are transcriptionally regulated themselves remain for the most part unknown. We found that in primary human memory T helper lymphocytes, miR-150 was the most abundantly expressed miRNA, and its expression decreased drastically upon activation, suggesting regulatory roles. Constitutive *MIR150* gene expression required the RFX family of transcription factors, and its activation-induced down-regulation was linked to their reduced expression. By performing miRNA pull-down and sequencing experiments, we identified PDGFA-associated protein 1 (PDAP1) as one main target of miR-150 in human T lymphocytes. PDAP1 acted as an RNA-binding protein (RBP), and its CRISPR/Cas-9–mediated deletion revealed that it prominently contributed to the regulation of T-cell proliferation. Overall, using an integrated approach involving quantitative analysis, unbiased genomics, and genome editing, we identified RFX factors, miR-150, and the PDAP1 RBP as the components of a regulatory axis that restrains proliferation of primary human T lymphocytes.

## Abstract 2

MicroRNAs exert key regulatory functions in the immune system, but their targets are largely unknown. This study shows that the ability of primary human T lymphocytes to proliferate in response to T cell receptor activation is modulated by a network comprising miR-150, transcription factors of the RFX family, and the RNA-binding protein PDAP1.

## Introduction

Through their ability to target a variety of mRNAs and regulate their translation and stability, microRNAs (miRNAs) modulate all aspects of the biology of T lymphocytes, including cell differentiation, activation, and proliferation [1,2]. The effect of any given miRNA is dependent on its expression level relative to that of its targets [3,4] and also on the specific context and cell-specific usage of target sites in the 3′ untranslated region (UTR) of mRNAs [5], resembling the cell type–specific regulation of gene expression mediated by transcription factors. The quantitative analysis of miRNA expression in different T-cell subsets and in response to T cell receptor (TCR) triggering may thus provide clues on the functional impact of individual miRNAs on T-cell responses. Abundant miRNAs that are down-regulated after stimulation may be involved in restraining T-cell activation, as shown in the case of miR-125b, which is required to maintain the naive state of human T cells [6]. By contrast, miRNAs that are expressed at very low levels are highly unlikely to reach the concentrations required to exert biological functions [4,7]. Finally, modestly expressed but inducible miRNAs may dynamically reach intracellular concentrations relevant in the modulation of T-cell activation. Examples in this group include miR-155 [8,9] and miR-146a [10], which are responsible for enhancing and attenuating T-cell responses, respectively.

In this study, we took advantage of an integrated approach combining quantitative miRNA analysis, unbiased genomics, and genome editing to identify miRNAs highly expressed in primary human T lymphocytes, analyze the regulatory logic underpinning their expression, and finally characterize mRNA target regulation and their functional impact.

Specifically, we focused on the single miRNA accounting for almost 50% of miRNAs constitutively expressed in human T cells, miR-150-5p (hereafter miR-150). This miRNA is abundantly expressed in both T and B lymphocytes [11], and its deletion in mouse models revealed that it modulates B lymphocyte and CD8^+^ T-cell differentiation [12–15]. To identify the mechanisms controlling constitutive miR-150 expression and its activation-induced down-regulation, we used an unbiased genomic approach to map the *cis*-regulatory elements in the *MIR150* locus that controlled its expression, leading to the identification of regulatory factor X (RFX) transcription factors as crucial regulators of constitutive miR-150 expression in resting cells and stimulus-induced down-regulation. Finally, we used miRNA pull-down and sequencing to identify the mRNAs specifically targeted by miR-150 in human T lymphocytes. MiR-150 targeted modulators of T-cell proliferation, including the transcription factor MYB and a previously unidentified target, PDGFA-associated protein 1 (PDAP1), which we characterized as an RNA-binding protein (RBP). Deletion of *MYB*, *PDAP1*, or *MIR150* itself by CRISPR/Cas-9–mediated gene editing in primary human T lymphocytes revealed the contribution of each of these factors to the regulation of T-cell proliferation in response to activating signals. Overall, our data identified a miRNA-regulated network involved in restraining proliferative responses of circulating resting T lymphocytes.

## Results

### MiR-150 is the most highly expressed miRNA in human T cells and is down-regulated by activation

**Fig 1:**
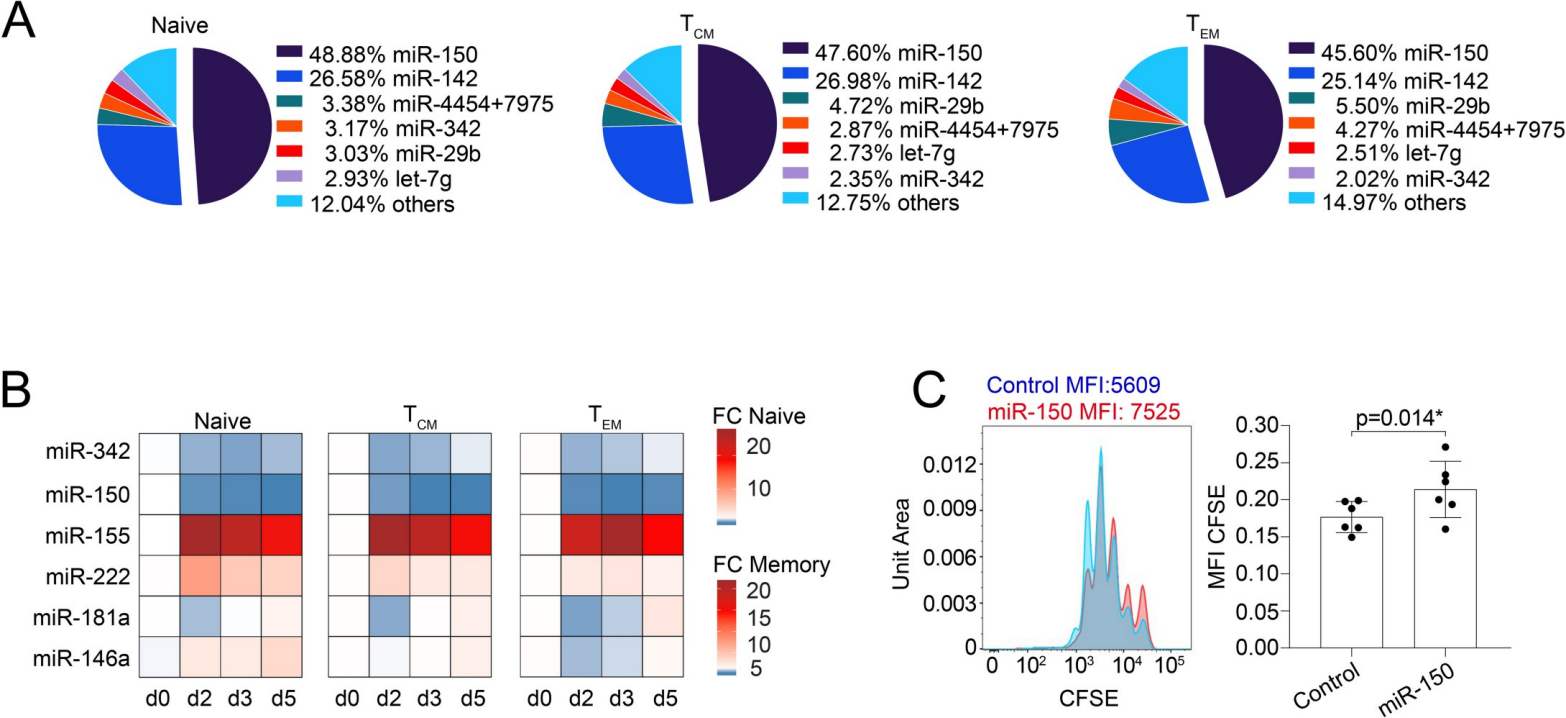
MiRNA expression in human CD4^+^ T-cell subsets. **(A)** Total RNA was extracted from freshly isolated CD4^+^ naive, T_CM_, and T_EM_ T-cell subsets, and miRNA expression was measured by NanoString SPRINT profiling. The most highly expressed miRNAs are shown, and data are expressed as percentage of normalized counts over the total. *N* = 3 independent donors. **(B)** Total RNA was extracted from the indicated T-cell subsets freshly isolated from peripheral blood. MiRNA expression was measured by RT-qPCR, and data are expressed as 2^−ΔCt^. *N* = 3 independent donors. **(C)** Freshly isolated memory T lymphocytes were loaded with CFSE, transfected with either a miR-150 mimic or a control oligonucleotide, and activated with anti-CD3 and anti-CD28 antibodies. The extent of cell proliferation was measured 3 days after activation. Data in the bar graph were normalized to the overall baseline signal on day 0, prior to stimulation, to compensate from experimental differences in basal CFSE loading. *N* = 6 independent experiments. Mean ± SD. Student *t* test, 2 tailed, paired. Underlying data can be found in S1 Data. CFSE, carboxyfluorescein succinimidyl ester; miRNA, microRNA; RT-qPCR, reverse transcription quantitative PCR.

To identify and accurately quantify miRNAs that are expressed by ex vivo isolated primary human T cells, we performed NanoString digital profiling of CD4^+^ naive, central memory (T_CM_), and effector memory (T_EM_) T-cell subsets isolated from 4 independent donors. Among the 827 miRNAs quantified, only 48 were detectable in these subsets (**S1 Table**). The levels of expression of these miRNAs differed widely, with the combined expression of only 2 of them (miR-150 and miR-142) representing >70% of the overall miRNA content in all the T-cell subsets analyzed (**Fig 1A**). MiR-150 was the most highly expressed miRNA, with an average number of approximately 110,000 molecules per 100 ng of total RNA (**S1A Fig**). While miR-150 expression was substantially similar among subsets, a few moderately expressed miRNAs (such as miR-222) were preferentially expressed in memory T cells (both T_CM_ and T_EM_) compared to naive cells, while miR-181a was instead preferentially expressed in naive compared to memory T lymphocytes (**S1B Fig**). No significant differences were observed between T_CM_ and T_EM_ cells (**S1B Fig**).

Next, we selected some of the highly expressed or differentially expressed miRNAs to assess their regulation in response to T-cell activation. T cells were stimulated with plate-bound anti-CD3 and anti-CD28 antibodies, and miRNA expression was measured by reverse transcription quantitative PCR (RT-qPCR) over time (**Fig 1B**). Some of the miRNAs expressed at moderate levels in resting lymphocytes (miR-155, miR-222, and miR-146a) were substantially induced upon TCR stimulation, especially in naive cells. Abundant miRNAs such as miR-150 and miR-342 were instead markedly reduced after 2 days of activation, while miR-181a had a more variable pattern of expression across the different subsets and time points. We further measured the expression of these highly abundant or inducible miRNAs in different ex vivo isolated effector subsets, namely T_H_1, T_H_2, T_H_17, and T_H_22 cells. We observed quantitatively modest and nonsignificant differences, concordant with differential miRNA expression being limited primarily to naive versus memory cells (**S1C Fig**). The dynamic regulation of miR-150 upon activation together with its high levels of expression in resting cells pointed toward its possible role in the regulation of T-cell responses upon TCR triggering.

To determine the functional role of miR-150 in human T cells, we transfected freshly isolated memory T lymphocytes with either a miR-150 mimic or a control oligonucleotide, and we measured cell proliferation over time by carboxyfluorescein succinimidyl ester (CFSE) dilution. We found that in the presence of miR-150, T-cell proliferation was significantly affected after 3 days of anti-CD3 and anti-CD28 stimulation, as shown by the reduced dilution of CFSE, leading to higher mean fluorescence intensity (MFI) (**Fig 1C**). Proliferation (measured by BrdU (bromodeoxyuridine) incorporation) was similarly reduced in Jurkat T cells stably transduced with a miR-150–expressing lentivirus (**S1D Fig**). Overall, miR-150 was the most highly expressed miRNA in human T lymphocytes, in which it controlled proliferation in response to stimuli.

### Identification of miR-150 targets in human T cells

**Fig 2:**
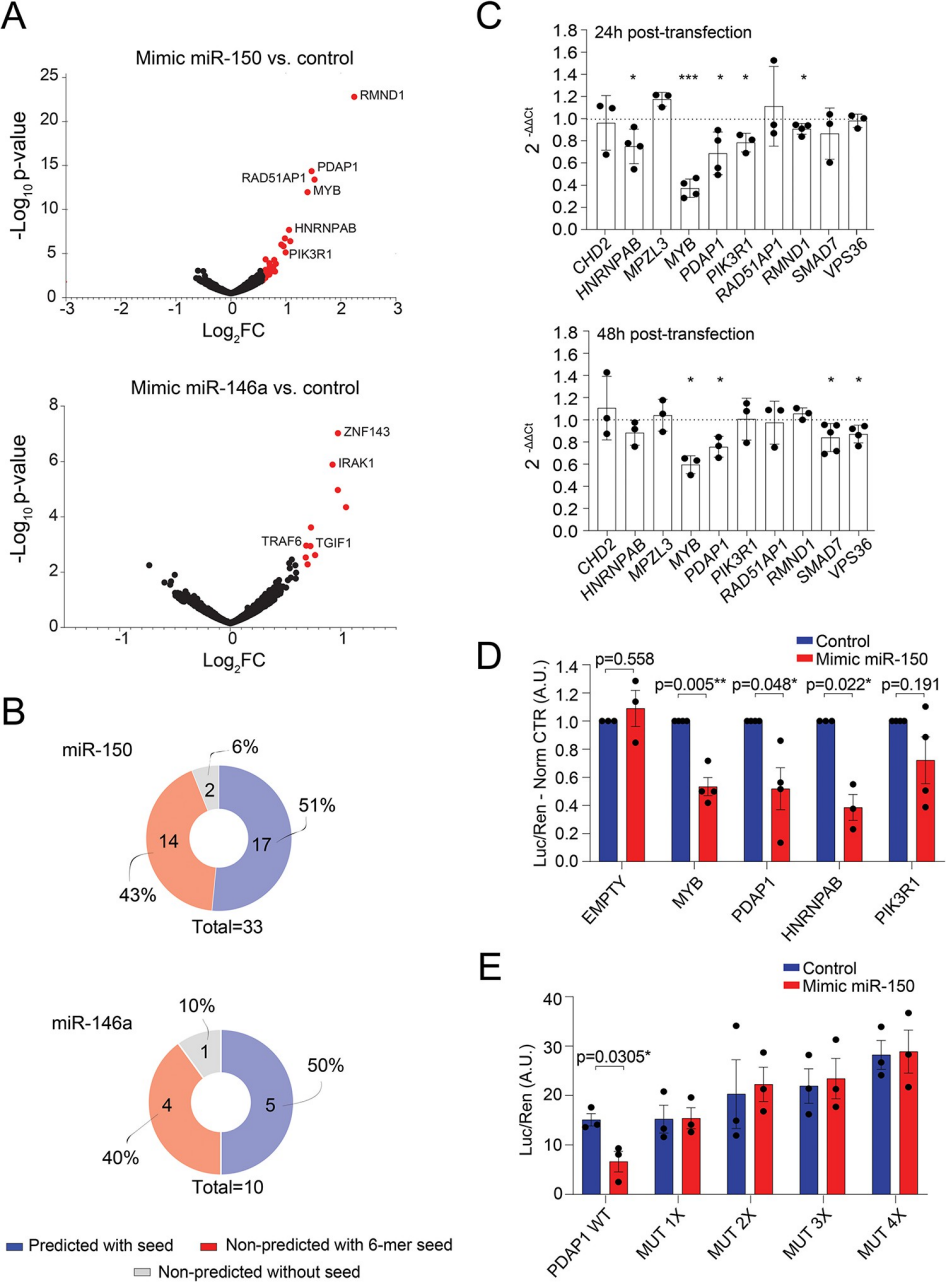
Identification of miR-150 targets. **(A)** Volcano plot of differentially expressed genes between the indicated miRNA mimic and control oligonucleotides. Genes in red were considered significantly differentially expressed when log_2_FC ≥ 0.6, −log_10_ *p*-value ≥ 2. **(B)** The list of genes obtained from (A) was intersected with several prediction databases using miRWalk 2.0. Both the 3′ UTRs and the CDS were manually searched for the presence of at least a 6-mer miR-150 or miR-146a binding site. **(C)** Activated memory cells were transfected with miR-150 mimic or control oligonucleotide. Twenty-four hours or 48 hours after transfection, the expression of the indicated genes was measured by RT-qPCR. *N* = 3 to 5 independent donors. Mean ± SD. Student *t* test, 2 tailed, paired. **(D)** The 3′ UTR of the indicated genes was cloned in a dual-luciferase reporter vector and transfected into HEK293 cells together with either a miR-150 mimic or a control oligonucleotide. Luciferase reads were normalized to the renilla ones. *N* = 3 to 4 independent experiments. Mean ± SEM. Student *t* test, 2 tailed, paired. **(E)** Same as in (D), except that the 4 putative miR-150 binding sites identified in the *PDAP1* 3′ UTR were mutated by site-directed mutagenesis. *N* = 3 independent experiments. Mean ± SEM. Student *t* test, 2 tailed, paired. Underlying data can be found in S1 Data. A.U., arbitrary unit; CDS, coding sequence; FC, fold change; miRNA, microRNA; RT-qPCR, reverse transcription quantitative PCR; UTR, untranslated region; WT, wild-type.

Cellular context–dependent regulation is a crucial aspect of miRNA-mediated regulation that is mainly based on the relative abundance of a miRNA and its targets within a specific cell type or activation state [5,16]. Such context-dependent regulation mediated by miRNAs cannot be predicted by the available databases and can only be experimentally explored. To identify the mRNAs that are directly and specifically regulated by miR-150 in T lymphocytes, we transfected activated memory T cells from 3 independent donors with either a biotinylated version of a miR-150 mimic or a control oligonucleotide, followed by streptavidin agarose pull-down and sequencing [17–20]. As a control of target specificity, we performed the same experiment using biotinylated miR-146a. We found that the pull-down of both miR-150 and miR-146a recovered established targets for these miRNAs, namely *MYB* for miR-150 and *IRAK1* and *TRAF6* for miR-146a, thus confirming target specificity (**Fig 2A**, **S2 and S3 Tables**). Further analysis of the recovered targets showed that 31 out of the 33 miR-150 putative targets contained at least one 6-mer seed within either the 3′ UTR, 5′ UTR, or the coding sequence (CDS), and approximately 50% (17 out of 33) of these were predicted miR-150 targets by the miRWalk 2.0 database [21] (**Fig 2B**). Our results are in line with previous observations, reported by other groups, showing that about half of bound miRNA sites are noncanonical, and that most noncanonical sites are bound and functional in a cell type–specific manner [5,22,23]. To which extent these noncanonical sites (that are efficiently bound in vivo) mediate effective target repression remains to be fully understood [24]. Similar results were obtained for miR-146a (**Fig 2B**).

Next, as validation of these pull-down data, we selected 10 putative miR-150 targets and tested the effects of a miR-150 mimic on their expression in T cells from an independent set of donors. Memory T cells were transfected with either the miR-150 mimic or control oligonucleotide, and mRNA expression was analyzed 24 hours or 48 hours later (**Fig 2C**). For some of the targets (*HNRNPAB*, *MYB*, *PDAP1*, *PIK3R1*, and *RMND1*), a suppressive effect of miR-150 was already observed after 24 hours, while for others (*SMAD7* and *VPS36*), a significant reduction was observed only after 48 hours, most likely due to varying mRNA stability and turnover. To determine whether the observed down-regulation of these putative miR-150 targets was mediated by a direct activity of miR-150 on their 3′ UTRs, we cloned either the entire 3′ UTR or the regions containing the predicted miR-150 binding site(s) in a reporter vector. Cotransfection of these plasmids with a miR-150 mimic oligonucleotide led to significantly reduced luciferase expression for 3 out of 4 targets tested, namely *MYB*, *PDAP1*, and *HNRNPAB*, which were therefore the highest confidence targets, while the effect on *PIK3R1* appeared to be more variable (**Fig 2D**). The *PDAP1* 3′ UTR contains 5 putative miR-150 binding sites predicted by TargetScan 7.2 [25], 4 of which are clustered in the distal region of the 3′ UTR. We cloned the region containing the 4 clustered sites, and we evaluated the impact of mutating these sites on miR-150–mediated repression. We found that mutation of only one site was sufficient to abrogate repression by miR-150, suggesting that all 4 clustered sites are required for full miR-150 activity on the *PDAP1* 3′ UTR (**Fig 2E**), although this effect may be different in vivo. To further investigate the relationship between PDAP1 and miR-150 in a more physiological setting, we deleted 1 or 3 clustered miR-150 binding sites from the 3′ UTR of the *PDAP1* gene in primary human T lymphocytes, using CRISPR/Cas-9 editing. We found that deletion of one single site was insufficient to completely abrogate miR-150 activity, while the partial deletion of 3 sites reduced miR-150 responsiveness (**S2 Fig**). Overall, our target analysis in primary human T lymphocytes recovered established targets of miR-150, such as *MYB*, and identified additional ones, such as *PDAP1*, as direct miR-150 targets in human T cells.

### PDAP1 acts as an RBP

**Fig 3:**
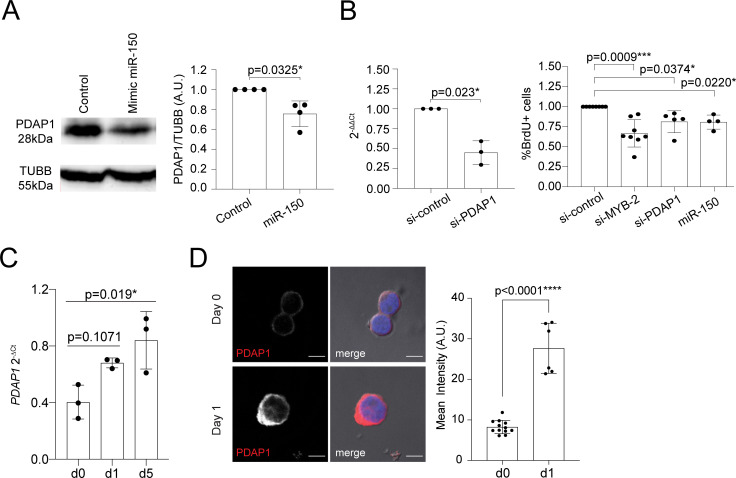
PDAP1 regulates T-cell proliferation and is up-regulated upon activation. **(A)** Memory T lymphocytes were transfected with either a miR-150 mimic or control oligonucleotide. Twenty-four hours after transfection, expression of PDAP1 was assessed by western blot. Tubulin was used as loading control. A representative western blot is shown on the left, while the densitometric quantification of independent experiments in shown on the right. *N* = 4. Mean ± SD. Student *t* test, 2 tailed, paired. **(B)** Memory T lymphocytes were transfected with siRNAs targeting either *MYB* or *PDAP1* or with a miR-150 mimic oligonucleotide. The extent of *PDAP1* down-regulation was measured by RT-qPCR (left), while cell proliferation was measured by BrdU incorporation assay. *N* = 3 to 8 independent experiments (each dot represents one donor). Mean ± SD. Student *t* test, 2 tailed, paired. **(C)** Memory T lymphocytes were stimulated with plate-bound anti-CD3 and anti-CD28 for the indicated days, followed by RT-qPCR analysis of *PDAP1* expression. *N* = 3 (each dot represents one donor). Mean ± SD. One-way ANOVA. **(D)** Memory T lymphocytes were stimulated with plate-bound anti-CD3 and anti-CD28 for the indicated days, followed by immunofluorescence for PDAP1. The bar corresponds to 5 μm. Representative of *N* = 2 experiments. Right: Quantification of the mean intensity of the PDAP1 signal in individual cells. Mean ± SD. Unpaired *t* test, 2 tailed. Underlying data can be found in S1 Data. PDAP1, PDGFA-associated protein 1; RT-qPCR, reverse transcription quantitative PCR; siRNA, small interfering RNA.

Consistent with the fact that *MYB* is a target of miR-150 [12], we found that in the presence of miR-150, endogenous *MYB* mRNA expression was significantly reduced compared to the baseline in both transiently transfected primary memory T lymphocytes and transduced Jurkat cells (**S3A Fig**). As expected, the downmodulation of *MYB* expression using small interfering RNAs (siRNAs) was sufficient to limit human T-cell proliferation (**S3B Fig**). Next, we investigated the role of additional miR-150 targets in the regulation of T-cell proliferation. We focused on *PDAP1* as it was the second most affected target in T cells transfected with miR-150. PDAP1 is a highly conserved protein whose precise functional role and mechanism of action is largely unknown. PDAP1 was found associated with different types of cancers [26,27], hinting at a role in the regulation of cell proliferation. Indeed, T-cell tumors also show high expression of PDAP1 [28]. First, we transfected memory T lymphocytes with a miR-150 mimic oligonucleotide, which led to a significant reduction in endogenous PDAP1 protein expression (**Fig 3A**).

Next, we investigated the impact of PDAP1 on memory T-cell proliferation using transfection of siRNAs. Compared to cells transfected with a control oligonucleotide, down-regulation of *MYB* or *PDAP1* or transfection of the miR-150 mimic oligonucleotide all led to a significant reduction in T-cell proliferation, as assessed by BrdU incorporation assay (**Fig 3B**). Consistent with a positive role of PDAP1 in controlling T-cell proliferation, we found that its expression increased, both at the mRNA and protein level, upon activation of T lymphocytes (**Fig 3C and 3D**). We also found that PDAP1 remained strictly cytoplasmic in both resting and activated cells, pointing toward a role in signaling and/or mRNA translation (**Fig 3D**).

**Fig 4:**
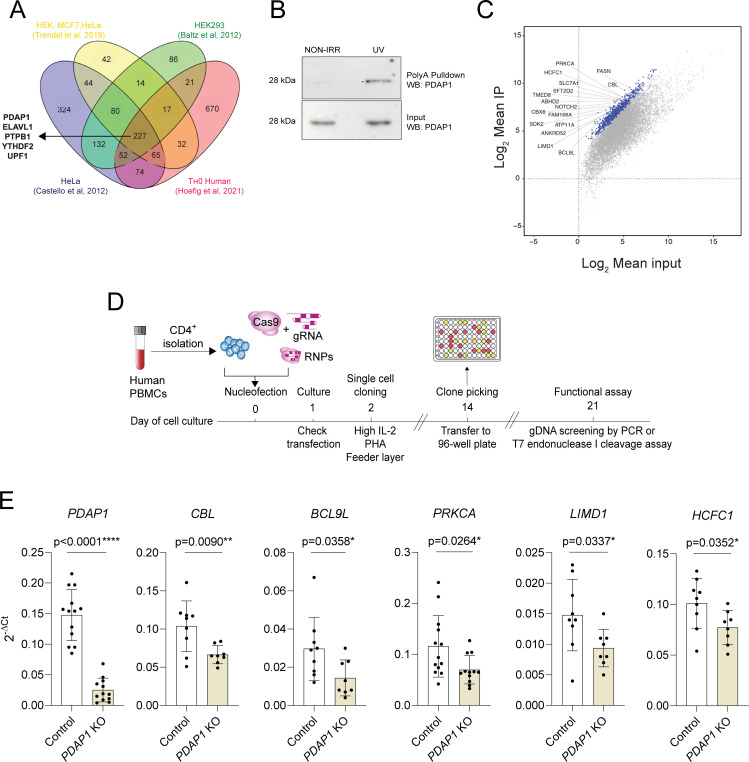
PDAP1 acts an RBP in primary human T lymphocytes. **(A)** Venn diagram showing the extent of overlap between RBPs identified in the indicated studies and cell types. **(B)** Activated memory T lymphocytes were UV-irradiated and lysed prior to poly-A mRNA pull-down and western blot. Nonirradiated samples were also used. Representative of *N* = 2 independent experiments. **(C)** Scatterplot of RIP-seq data (*N* = 3 independent donors). Blue dots indicate transcripts significantly enriched (FDR < 0.001) in RIP samples compared to input controls, with log_2_FC ≥ 2, as determined by DESeq2. All other mRNAs are indicated as gray dots. **(D)** Schematic representation of the experimental workflow to generate primary T lymphocytes KO for a protein of interest. **(E)** Primary human CD4^+^ memory T lymphocytes were transfected with CRISPR/Cas-9 RNPs to delete *PDAP1*, followed by single-cell cloning and screening. Clones showing a prominent genomic deletion in the *PDAP1* gene were used to measure the expression of *PDAP1* itself and of other transcripts bound by PDAP1. Each dot represents one individual clone (at least *N* = 8). Statistical test is unpaired *t* test or Mann–Whitney, depending on the distribution of the clones. Mean ± SD. Underlying data can be found in S1 Data. FDR, false discovery rate; FC, fold change; gDNA, genomic DNA; gRNA, guide RNA; KO, knockout; PBMC, peripheral blood mononuclear cell; PDAP1, PDGFA-associated protein 1; RBP, RNA-binding protein; RIP-seq, RNA immunoprecipitation sequencing; RNP, ribonucleoprotein.

The mechanisms by which PDAP1 regulates proliferation is incompletely understood, although it was recovered as an RBP in several RNA–protein interactome studies [29–32], and it was also described as an RNA-dependent protein, namely a protein able to engage in larger, yet uncharacterized, complexes only in the presence of RNA [33]. Indeed, PDAP1 was reproducibly recovered after RNA pull-down in different human cell types, including human T lymphocytes [29–32], pointing toward a crucial function of this protein as an RBP (**Fig 4A, S4 Table**). To experimentally determine whether PDAP1 can indeed act as an RBP in human T lymphocytes, we performed an oligo-dT pull-down of total mRNA in memory T cells. Western blot analysis of these samples revealed the presence of PDAP1, which was preferentially enriched in samples that underwent UV crosslinking (**Fig 4B**; raw data in **S4 Fig**), suggesting that this protein is indeed capable of RNA binding in T cells, either directly or as part of a larger RNA-binding complex, yet to be determined.

Next, we searched for candidate targets that might be regulated by this protein. RNA immunoprecipitation (RIP) and sequencing experiments in UV-crosslinked memory T lymphocytes using an anti-PDAP1 antibody revealed several factors crucial in the regulation of T-cell biology that were bound by PDAP1, most prominently some regulators of T-cell activation, differentiation, and proliferation such as *CBL* [34], *NOTCH* factors [35], and the multiple sclerosis susceptibility gene *PRKCA* [36,37] (**Fig 4C, S5A Fig, S5 Table**). Many of the most highly significantly enriched transcripts (indicated in blue in **Fig 4C**) were expressed at moderate to high levels in human T cells as shown in the DICE database [38], although some others, like *SDK2*, were lowly expressed. To investigate the impact of PDAP1 expression on these bound transcripts, we considered the top 30 most enriched transcripts. We then selected 10 of them based on their potential involvement in modulating T-cell activation or cell cycle progression in different systems. Next, we generated primary T-cell clones in which the *PDAP1* gene was deleted by CRISPR/Cas-9, and we measured the levels of the selected transcripts (**Fig 4D and 4E, S5B Fig**). The workflow of the overall experimental design included transfecting primary human memory T lymphocytes with 2 single-guide RNAs (sgRNAs) for the gene of interest together with recombinant Cas9 protein, followed by single-cell cloning, expansion, selection of gene-modified clones, and functional analyses (**Fig 4D**) [39,40]. After transfection, primary memory T cells were cloned in 384-well plates by limiting dilution, after which individual clones were screened for the presence of insertions/ deletions (indels) or mutations in the genomic region of *PDAP1* by PCR or T7 endonuclease I cleavage assay (**S6 Fig**).

We found that the deletion of *PDAP1* significantly reduced the expression of *CBL* and other PDAP1-bound transcripts, such as *BCL9L* and *PRKCA* (**Fig 4E, S5B Fig**), pointing toward a required role of PDAP1 in modulating the stability of at least a subset of the bound transcripts. Expression of *LIMD1*, a component of P-bodies and involved in their formation and integrity [41], was also affected, further highlighting a possible link between PDAP1 and posttranscriptional regulation of mRNA stability. However, the expression of other bound transcripts remained unaffected (**S5B Fig**), which may suggest redundancy provided by other regulators or the involvement of PDAP1 in regulatory mechanism not related to mRNA stability (e.g., translational control). The transcripts for *PDAP1* itself and *MYB* were not significantly enriched by PDAP1 immunoprecipitation. Interestingly, knockdown of *PDAP1* also modestly affected the expression of *MYB* (**S5C Fig**), although this could be an indirect effect due, for instance, to the altered availability of miR-150 upon removal of one its abundantly expressed primary targets. Even though the mechanistic underpinning of PDAP1 function and regulation on mRNA stability or translation remains to be understood, these results further indicate that PDAP1 is an RBP capable to modulate T-cell proliferation at least in part by affecting the expression of factors that are central to T-cell activation and metabolism.

### Deletion of PDAP1 limits lymphocyte proliferation

**Fig 5:**
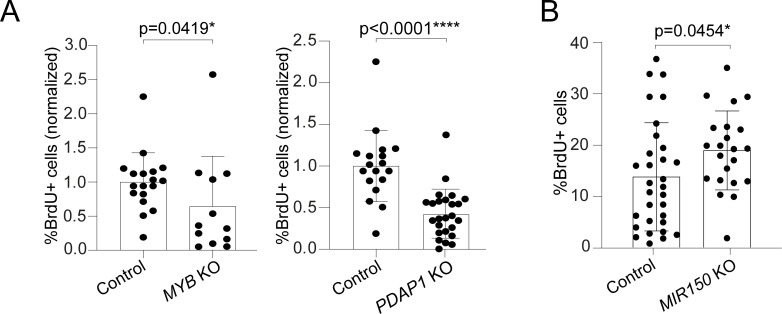
miR-150 restrains T-cell proliferation through MYB and PDAP1. **(A)** Primary memory T cells were transfected with Cas-9 RNPs to delete either *MYB* (left) or *PDAP1* (right), followed by single-cell cloning. Individual clones were selected based on the presence of a large genomic deletion in the gene of interest, and proliferation was measured by BrdU incorporation assay. For the *MYB* gene, *N* = 18 control clones and *N* = 12 *MYB*-edited clones, from 2 independent donors. For the *PDAP1* gene, *N* = 18 control clones and *N* = 25 *PDAP1*-edited clones, from 2 independent donors. Mean ± SD. Mann–Whitney test. **(B)** Memory T cells were transfected with Cas-9 RNP complexes containing 2 different sgRNAs targeting the *MIR150* gene. Individual clones were selected based on the presence of a genomic deletion overlapping the *MIR150* sequence, and proliferation was measured by BrdU incorporation assay. *N* = 31 control clones and *N* = 22 *MIR150*-edited clones, from 2 independent donors. Mean ± SD. Welch *t* test, 2 tailed. Underlying data can be found in S1 Data. sgRNA, single-guide RNA; KO, knockout; PDAP1, PDGFA-associated protein 1; RNP, ribonucleoprotein.

To further assess the role of miR-150 and its targets in T-cell proliferation, we performed CRISPR/Cas-9–mediated deletion of either the *PDAP1* or *MYB* gene (**S6A and S6B Fig**). We found that the cloning efficiency in 3 independent donors was on average 16% for PDAP1 and 15% for MYB, compared to 24% for the control clones (transfected with sgRNAs targeting an irrelevant, non-expressed gene), suggesting that the targeted genes affected the ability of the clones to expand. Cell proliferation of individual clones was therefore measured by BrdU incorporation. By analyzing 12 *MYB*-KO and 25 *PDAP1*-KO clones, we found that T-cell proliferation was significantly decreased for both (**Fig 5A**). A similarly reduced proliferation was observed also in Jurkat cells transfected with sgRNAs against either *MYB* or *PDAP1* (**S6D Fig**).

Further highlighting the role of PDAP1 in modulating lymphocyte proliferation, we found that in a panel of B cell lymphoma cell lines, PDAP1 expression was often increased compared to primary B lymphocytes, and CRISPR/Cas-9 deletion of PDAP1 in these cell lines led to significantly reduced proliferation (**S7 Fig**). Finally, to unequivocally determine whether miR-150 expression was sufficient to restrain T-cell proliferation, we transfected memory T cells with 2 sgRNAs targeting the *MIR150* gene (**S6C Fig**), which led to high deletion efficiency (67%). We found that upon targeting the *MIR150* locus, T cells from 2 independent donors proliferated significantly more (approximately 37% increase) compared to control clones (**Fig 5B**). Overall, the experimental identification of miR-150 targets coupled to their functional validation revealed that PDAP1 is a crucial regulator of T-cell proliferation whose activity is restrained in the resting state by high levels of miR-150.

### RFX family transcription factors modulate miR-150 expression

**Fig 6:**
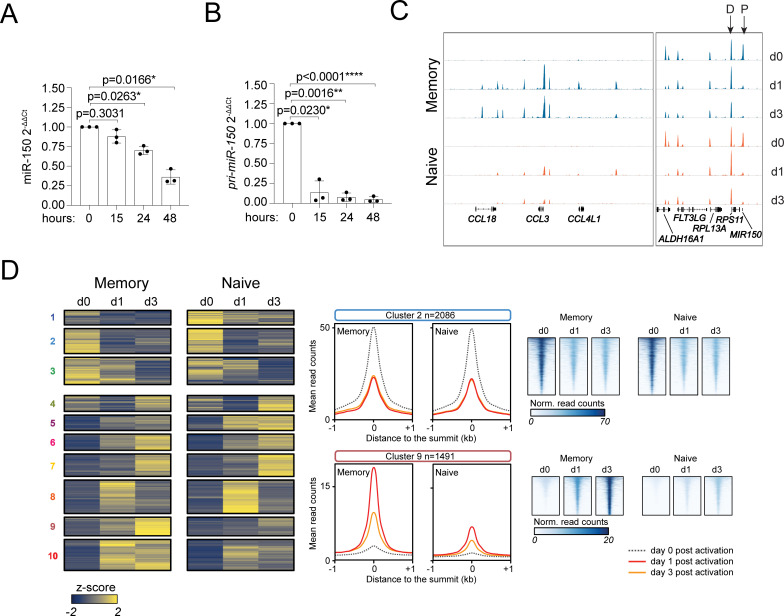
ATAC-seq analysis in human CD4^+^ T-cell subsets. **(A)** Memory T cells were sorted from peripheral blood and were either left resting or activated with plate-bound anti-CD3 and anti-CD28 antibodies for the indicated number of hours. Total RNA was extracted and miR-150 expression was measured by RT-qPCR. *N* = 3 independent donors (each dot represents one donor). Mean ± SD. One-way ANOVA. Underlying data can be found in S1 Data. **(B)** Same as (A), except that the expression of *pri-miR-150* was measured. *N* = 3. Mean ± SD. One-way ANOVA. Underlying data can be found in S1 Data. **(C)** Naive and memory T cells were freshly isolated from 3 independent donors and were either left resting or were stimulated with anti-CD3 and anti-CD28 antibodies for 1 or 3 days, before tagmentation and sequencing for ATAC-seq analysis. Representative snapshots of the sequencing tracks. Arrows indicate the distal (D) and proximal (P) peaks relative to *MIR150*. **(D)** Clustering analysis including all ATAC-seq peaks that were significantly affected after 24 hours or 72 hours of activation in either naive or memory T cells, with different representations of cluster 2 and 9 (middle and right panels). ATAC-seq, assay for transposase accessible chromatin and sequencing; RT-qPCR, reverse transcription quantitative PCR.

High levels of miR-150 in basal conditions were coupled with its strong reduction upon T-cell activation. In memory T cells, the reduced abundance of miR-150 was significant already at 24 hours of stimulation with anti-CD3 and anti-CD28 antibodies (**Fig 6A**), namely before cells started to proliferate [42], thus ruling out a role of passive dilution of the mature miRNA. To investigate potential mechanisms of miR-150 down-regulation, we first measured the expression of the primary (*pri-miR-150*) transcript. We found that its expression was almost completely abrogated 15 hours after activation (**Fig 6B**), pointing toward the rapid down-regulation of *MIR150* gene transcription upon T-cell activation.

To identify in an unbiased manner the transcriptional mediators of miR-150 down-regulation in activated primary T lymphocytes, we first set out to identify the genomic *cis*-regulatory elements in the *MIR150* locus that were deactivated upon stimulation. To this aim, we performed assay for transposase accessible chromatin and sequencing (ATAC-seq) in primary human naive and memory T cells, either resting or activated with anti-CD3 and anti-CD28 antibodies for 1 or 3 days. In memory T cells, 747 peaks were already significantly reduced after 1 day of activation (log_2_ fold change ≤ −1 and adjusted *p*-value ≤ 10^−5^). Of these, 103 peaks could be associated to a transcription start site (TSS). In the same samples, 6,237 peaks instead increased after activation. Naive T cells showed similar results, with 923 ATAC-seq peaks reduced after activation, with 124 of them matching a TSS and 6,616 induced peaks (**S6 Table**).

Visual inspection of the data revealed 2 prominent ATAC-seq peaks in the proximity of the *MIR150* gene (**Fig 6C**, right panel): The distal one (D) was located upstream of *MIR150* and was likely to be involved in the regulation of the adjacent gene *RPS11*, but a role in the control of *MIR150* itself cannot be ruled out since the promoter and TSS of the full *pri-miR-150* gene remain undetermined. This peak showed modest, if any, changes during T-cell activation and corresponded to a region bound by the transcription factor XBP1 in T lymphocytes [43].

The second, proximal peak (P) almost perfectly coincided with the miR-150 sequence (**Fig 6C**, right panel), and while it was very prominent in both naive and memory resting T lymphocytes, it quickly and almost completely disappeared in both cell subsets 24 hours after stimulation, and it was not regained at a later time point, suggesting a possible role in the direct control of *MIR150* expression.

In a clustering analysis including all ATAC-seq peaks that were significantly affected after 24 hours or 72 hours of activation in either naive or memory T cells (*n* = 16,697 sites), the down-regulated peak overlapping *MIR150* belonged to a large cluster (cluster 2, **Fig 6D**) that included all peaks that lost accessibility after 1 day of stimulation in both naive and memory T cells, hinting at a shared regulation associated with activation. Other clusters included peaks that were more gradually reduced over time (clusters 1 to 4) or those that were variably induced by activation (clusters 5 to 10) (**S8 Fig**). Several peaks, like those in cluster 9, were more strongly affected in memory compared to naive T cells, pointing toward regulatory regions and genes likely to be more active in one of the subsets (**Fig 6D**).

**Fig 7:**
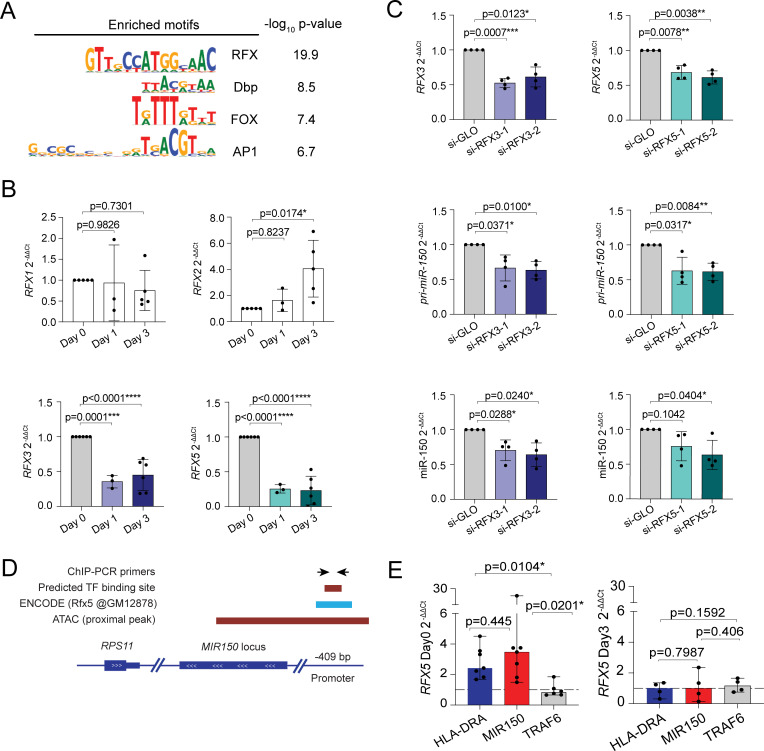
RFX factors regulate miR-150 expression. **(A)** Transcription factor motifs enrichment analysis. Peaks in cluster 2 were compared to all accessible sites detected. **(B)** Memory T lymphocytes were stimulated with plate-bound anti-CD3 and anti-CD28 antibodies for the indicated times. Total RNA was extracted and the expression of the different *RFX* mRNAs was measured by RT-qPCR. *N* = 3 to 6 independent donors (each dot represents one donor). Mean ± SD. One-way ANOVA. **(C)** Resting memory T lymphocytes were transfected with siRNAs targeting either *RFX3* (left) or *RFX5* (right). Twenty-four hours after transfection, total RNA was extracted and the expression of the indicated genes measure by RT-qPCR. *N* = 4 independent donors (each dot represents one donor). Mean ± SD. Paired *t* test, 2 tailed. **(D)** Schematic representation of the *MIR150* locus, with overlapping ATAC peak and the location of an RFX5 ChIP-seq peak in the human B lymphocyte cell line GM12878 as described by the ENCODE project (ChIP-Atlas). The location of PCR primers for ChIP analysis of RFX5 binding is also indicated. **(E)** RFX5 binding at the indicated genomic loci was determined by ChIP-qPCR in resting (day 0) and activated (day 3) memory T lymphocytes. Data were normalized on the input and a control immunoprecipitation with an irrelevant antibody for each target (dashed line y = 1). Target genes exceeding the dashed line threshold were considered to be bound by RFX5. Data are shown as median with 95% confidence interval; at least *N* = 4 independent human donors. Each dot represents one experiment. Ratio paired *t* test, 2 tailed. Underlying data can be found in S1 Data. ChIP, chromatin immunoprecipitation; RT-qPCR, reverse transcription quantitative PCR; RFX, regulatory factor X; siRNA, small interfering RNA.

We subsequently focused on cluster 2, containing the ATAC-seq peak coinciding with *MIR150*. In order to identify transcription factor DNA binding motifs associated with the accessible regions in this cluster, we performed transcription factor motif enrichment analysis [44]. To this aim, peaks in cluster 2 were compared to all accessible sites detected. The DNA binding motifs recognized by the RFX family of transcription factors were consistently the sites most overrepresented in cluster 2 in both naive and memory cells (**Fig 7A, S7 Table**).

Among the 8 members of the RFX family, RFX4, RFX6 and RFX8 showed low to undetectable expression in T lymphocytes according to both the Human Protein Atlas [45] and the Database of Immune Cells (DICE) [38]. RFX2 was also lowly expressed. We therefore assessed the expression of the remaining RFX family members in resting and activated naive and memory T cells. We found that *RFX7* expression was diminished in naive T cells upon activation but induced in memory cells (**S9A Fig**), while expression of *RFX1* did not change significantly upon T-cell activation, and *RFX2* expression increased over time (**Fig 7B, S9B Fig**), all patterns that were not consistent with the rapid down-regulation of miR-150 expression in both naive and memory T cells. Conversely, expression of both *RFX3* and *RFX5* strongly diminished upon activation (**Fig 7B, S9B Fig**), hinting at their potential involvement in the regulation of miR-150 expression. To determine the functional impact of RFX3 and RFX5 on miR-150 expression, we performed RNA interference (RNAi) experiments in primary resting memory T cells. We found that down-regulation of either *RFX3* or *RFX5*, as determined by RT-qPCR reduced the expression of *pri-miR-150* and mature miR-150 (**Fig 7C**). In these experimental conditions, the levels of miR-150 remain, however, overall very high; therefore, no significant effect on *MYB* and *PDAP1* expression could be measured (**S9C Fig**). Finally, overexpression of *RFX3* or *RFX5* in T lymphocytes before activation did not significantly affect miR-150 expression or T-cell proliferation after 5 days of activation (**S10 Fig**). Therefore, RFX3 and RFX5 are both required to maintain basal miR-150 expression in resting T cells, but they are not sufficient to avoid the drastic reduction in miR-150 expression that occurs upon TCR activation.

Next, we explored whether such regulation was due to direct RFX factor binding to the *MIR150* locus. Browsing of the ChIP-Atlas (chip-atlas.org [46]) identified an RFX5 ChIP-seq (chromatin immunoprecipitation sequencing) peak in B lymphocytes upstream of the miR-150 sequence (GM12878 ENCODE [47]), within the ATAC-seq peak identified in our dataset (**Fig 7D**). To assess whether RFX5 directly bound the *MIR150* locus in T lymphocytes, we performed RFX5 ChIP-qPCR in primary human CD4^+^ T lymphocytes either resting or activated with anti-CD3 and anti-CD28 antibodies for 3 days. As a positive control for RFX5 binding, we used a region of the *HLA-DRA* gene containing an RFX5 binding site [48]. We found that in resting, but not in activated T cells, the region containing the *MIR150* locus was enriched in the RFX5-bound DNA fraction, while no enrichment was detected for the nontarget gene *TRAF6* (**Fig 7E**). Thus, RFX5 regulated basal miR-150 expression in resting T cells by binding directly to the *MIR150* locus.

Overall, we identified a regulatory network required to restrain lymphocyte proliferation, composed by an RFX-miR-150 axis required to limit the activity of factors important for T-cell proliferation, most notably the RBP PDAP1.

## Discussion

In this work, we identified RFX family members as factors involved in maintaining basal miR-150 expression in resting cells, which, in turn, restrained proliferation by targeting *MYB* and *PDAP1*. The role of miR-150 in the control of cell proliferation is suggested by several pieces of evidence. First, it is significantly down-regulated in different types of T-cell lymphoma, including peripheral T-cell lymphomas (PTCLs) and advanced cutaneous T-cell lymphoma (CTCL) [49,50]. Second, reduced miR-150 expression was associated with invasion and metastasis in mouse models, suggesting that in these cells, miR-150 acts as a crucial tumor suppressor.

While our analyses identified MYB and PDAP1 as direct miR-150 targets in activated human T lymphocytes, a few other targets previously identified in CD4^+^ T cells were not detected in our study. For instance, miR-150 was shown to cooperate with miR-99 to repress mTOR expression and to promote regulatory T cell (Treg) differentiation in the mouse. Interestingly, miR-150 could only exert its repressing activity on this target in the presence of miR-99, pointing toward cooperativity between different miRNA binding sites [51]. Other reported miR-150 targets included *AKT3* [52] and *SLC2A1* (GLUT1), which was targeted by miR-150 in regulatory Th1 cells stimulated with anti-CD3 and anti-CD46 antibodies [53]. The fact that these genes were not identified in our experiments might be linked to the different T-cell subsets under consideration or to the conditions of T-cell culture and stimulation. Moreover, for most other reported targets, the strength of a direct in vivo association with miR-150 was not assessed. In this respect, the pull-down approach that we used to identify T-cell–specific targets may also be limited by the necessity to balance stringency (to reduce false positives) and sensitivity, which might lead to the predominant enrichment of abundant targets that are most strongly regulated by miR-150 [17,19,54].

Apart from *MYB*, we identified *PDAP1* as a direct miR-150 target implicated in regulating T-cell proliferation. PDAP1 (also known as PAP, HAP28) is a 28-kDa phosphoprotein that was originally identified as a modulator of mitosis in association with PDGFA and PDGFB in rat neural retina cells [55]. Browsing of the Human Protein Atlas [45] revealed the broad tissue expression of PDAP1 and mainly cytoplasmic and plasma membrane–associated expression. However, very little is known about the physiological functions of this protein, although a genetic association with mendelian diseases of the nervous system was identified [56]. In T lymphocytes, RBPs such as the Roquin, Regnases, and the TTP family of proteins have an important role in posttranscriptional gene regulation, being key actors in modulating T-cell activation and functions, for instance, through the regulation of cytokine mRNA expression and stability [39,57]. These proteins often contain defined RNA-binding domains able to recognize specific features on the transcripts, such as AU-rich elements and stem–loop structures [57]. Although our own data and data from RNA-interactome studies clearly revealed that PDAP1 can act as an RBP, it contains no recognizable RNA-binding domains (D^2^P^2^, Database of Disordered Protein Predictions) [58], an observation compatible with the intrinsically disordered regions often observed in an abundant and understudied class of noncanonical RBPs [29,30,32,59]. Interestingly, although the *PDAP1* mRNA contains a CDS that measures only 546 nucleotides in length, its 3′ UTR is instead much larger (more than 2 kb), pointing toward a highly regulated expression for this protein mediated both by miRNAs as shown in this study and potentially also through extensive cross-regulation with other RBPs, as shown for many other instances of RBP regulation [57]. In murine B lymphocytes, PDAP1 was shown to protect mature B cells from stress and to favor antibody diversification, although no clear mechanism of action could emerge, most likely due to the high number of genes that were affected both positively and negatively in the absence of PDAP1 [60]. Such large changes in the transcriptome of cells lacking PDAP1 are likely to be the result of a complex pattern of direct and indirect effects. Furthermore, like binding of a transcription factor to target DNA does not necessarily result in transcription changes, binding of an RBP to a target may not result in changes in target abundance. Binding may, for instance, result in the sequestration of the target, making it less available to proteins regulating its translation and/or the bound protein may exert its functional effects in a signal-dependent manner, therefore undetectable in the absence of the appropriate stimulus. What is the exact mechanism of action of PDAP1 and to what extent its RNA-binding capacity is relevant to its functions remains to be understood, and it will be the subject of future studies. At this stage, we also have no evidence for any direct relationship between MYB and PDAP1, apart from both of these factors being targeted by miR-150 and being involved in regulating T-cell proliferation.

One other intriguing observation of our study is the very rapid reduction of miR-150 levels after T-cell activation. Other miRNAs, like let-7, were also reported to be down-regulated upon T-cell activation, at least in murine CD8^+^ T cells [61]. While we found that a large component of miR-150 down-regulation was transcriptional, some posttranscriptional mechanisms may also be at play and would probably contribute to explain the observed reduction of miR-150 expression even in the absence of cell division. For instance, miR-150 was shown to be degraded by the inositol-requiring enzyme 1α (IRE1α), which possesses endoribonuclease activity toward cellular mRNAs, which was shown to directly cleave selected miRNAs, including miR-17a and miR-150 [62,63]. Finally, miR-150 is highly abundant in extracellular vesicles derived from activated primary human T lymphocytes [64,65], a process that may also be important to achieve the rapid elimination from the cytoplasm of negative regulators of lymphocyte activation [64,66]. Apart from the abrupt loss of transcription described in our study, these posttranscriptional mechanisms may also variably contribute to the reduction of mature miR-150 in the cytoplasm, thereby allowing full-blown T-cell activation.

## Methods

### Isolation, culture, and activation of human CD4^+^ peripheral T lymphocytes

Buffy coats from healthy donors were obtained from the Swiss Blood Donation Center of Basel and Lugano (Switzerland), with informed consent from the Swiss Red Cross and authorization number CE 3428 from the Comitato Etico Canton Ticino. Leukocytes were separated by gradient centrifugation (Ficoll-Paque Plus; GE Healthcare, IL, USA), then CD4^+^ T cells were isolated by magnetic microbeads and LS columns (Miltenyi Biotec, Germany). Naive and memory T-cell subsets were then sorted using FACSaria (BD Bioscience, NJ, USA) based on the expression of the following surface markers: naive T cells: CD4^+^CD25^−^CD45RA^+^CCR7^+^; total memory T cells: CD4^+^CD25^−^CD45RA^−^CCR7^+/−^; T_CM_ cells: CD4^+^CD25^−^CD45RA^−^CCR7^+^; T_EM_ cells: CD4^+^CD25^−^CD45RA^−^CCR7^−^. Other T-cell subsets were separated from CD4^+^ T cells by sorting for the following surface markers: T_H_1 cells: CD4^+^CD25^−^CD45RA^−^CXCR3^+^CCR4^−^CCR6^−^; T_H_2 cells: CD4^+^CD25^−^CD45RA^−^CXCR3^−^CCR4^+^CCR6^−^; T_H_17 cells: CD4^+^CD25^−^CD45RA^−^CXCR3^−^CCR4^+^CCR6^+^; T_H_22 cells: CD4^+^CD25^−^CD45RA^−^CCR4^+^CCR6^+^CCR10^+^. When needed, cells were cultured in RPMI-1640 medium supplemented with 5% human serum, 1% nonessential amino acids, 1%, sodium pyruvate, 1% glutamine, penicillin, streptomycin, and 50-μM β-mercaptoethanol (complete medium). Cells were activated in Nunc MaxiSorp flat 96-well plates with plate-bound anti-CD3 (recombinant TR66 clone, in house production) and anti-CD28 (1 μg/mL) antibodies. Cells were removed from stimuli and placed in a round bottom plate after 48 hours. When needed, cultures were supplemented with IL-2 at the concentration of 60 U/mL after the initial 5 days of activation.

### Transfection of primary T cells and cell lines

Primary T cells were transfected with NEON nucleofector (Invitrogen, MA, USA) at 1,800 to 2,200 V, 20 ms, 1 pulse, using the provided buffer T. A total of 1 × 10^6^ cells was used with the 10-μl tip transfections or 2.5 × 10^6^ cells with the 100-μl tip. Locked nucleic acids (LNAs) and siRNAs (listed in **S8 Table**) were used at the concentration of 2 μM. Primary cells were cultured for up to 48 hours posttransfection in prewarmed complete medium without antibiotics. About 2.5 × 10^6^ Jurkat cells were nucleofected at 1,325 V, 10 ms, 3 pulses using buffer R and were cultured in RPMI-1640 medium supplemented with 10% FBS, 1% nonessential amino acids, 1% sodium pyruvate, 1% glutamine, penicillin, streptomycin, kanamycin, and 50-μM β-mercaptoethanol. HEK293 cells were transfected with polyethylenimine (PEI) using standard protocols and cultured in DMEM with 4.5 g/L D-glucose supplemented with 10% FBS, 1% sodium pyruvate, penicillin, streptomycin, and 50-μM β-mercaptoethanol.

### Lentivirus production and cell transduction

Lentiviral particles were purified from the supernatant of transfected HEK293 cells by sucrose gradient (10 mM Tris-HCl pH 7.5, 100 mM NaCl, 1 mM EDTA, 25% sucrose) and ultracentrifugation (2.5 hours, 100,000 × *g*, 4°C). For some experiments, lentivirus particles were concentrated using a PEG-8000 solution [67] (80 g PEG-8000, 14 g NaCl in 200 ml of PBS, pH 7.2) followed by centrifugation at 1,600 × *g*, 1 hour at 4°C. For primary T cells, 5 μl of lentiviral concentrate were mixed with 150,000 resting cells in a flat-bottom 96-well plate. After 24 to 48 hours, cells were transferred to a Nunc plate coated with anti-CD3 and anti-CD28 antibodies for 48 hours and finally transferred to a round bottom 96-well plate with until day 5 to 7. Jurkat T cells were transduced with 5 μl of lentivirus-containing medium in a 96-well plate, 150,000 cells/well. After 48 to72 hours, transduced cells were sorted for GFP expression.

### Plasmids and cloning

Plasmids were constructed and modified by standard molecular cloning techniques. The miR-150 lentiviral vector pLL3.7_hsa-miR-150 (#25792) [68] and the empty backbone pLL3.7 (#11795) [69] were obtained from Addgene (MA, USA). The RFX3- and RFX5-expressing lentiviral vectors were obtained from GeneCopoeia (MD, USA). For dual luciferase assays, regions of approximately 600 bp containing the putative miR-150 sites were amplified by PCR from the 3′ UTRs of the *MYB*, *PDAP1*, *HNRNPAB*, and *PIK3R1* genes and were cloned into the pmirGLO plasmid using the NheI/XbaI restriction sites. The *PDAP1*-3′ UTR-containing plasmid was further mutated to abrogate the miR-150 binding sites by site-directed mutagenesis using the Quick Change II kit (Agilent, CA, USA) according to manufacturer’s instructions. All plasmids were verified by Sanger sequencing.

### Luciferase assay

HEK293 cells were transfected in a 96-well plates with 25 ng of pmirGLO plasmid (containing part of the 3′ UTR of the candidate target downstream to luciferase gene) and 1 μM of miR-150 mimic or control oligonucleotide using a standard PEI protocol. After 24 hours, cells were lysed and analyzed using the Dual-Luciferase Reporter Assay System (Promega, WI, USA) and a GloMax Luminometer (Promega).

### NanoString SPRINT profiling

Total RNA was isolated using TRI reagent RT (MRC, OH, USA) and ZymoSpin columns and eluted in nuclease-free water. When needed, RNA was concentrated with a speed vac. Moreover, 100 ng of total RNA at a concentration of 33 ng/μl were used for each experimental condition and probed with the Human miRNA v3 assay, according to manufacturer’s instructions. Data were normalized to the 25 most highly expressed hits, and *p*-values and ratios were calculated using the n-Solver 3.0 software.

### RT-qPCR

Total RNA was isolated using TRI reagent RT (MRC) and ZymoSpin columns and eluted in nuclease-free water. For gene expression analysis, RNA was retrotranscribed with qScript cDNA SuperMix (Quanta Biosciences, MA, USA) and PCR performed with PerfeCTa SYBR green FastMix (Quanta Biosciences) using primers listed in **S8 Table**. For miRNA expression, TaqMan MicroRNA Reverse Transcription Kit (Applied Biosystems, MA, USA) and TaqMan Universal PCR Master Mix (Applied Biosystems) were used. Taqman probes are listed in **S8 Table**. PCR reactions were run on the ABI 7900HT Fast Real-Time PCR System (Applied Biosystems) or using the Quant Studio 3 Real-Time PCR System (Thermo Fisher Scientific, MA, USA). Data were normalized to the *UBE2D2* housekeeping gene for SYBR-based qPCRs or on RNU48 for Taqman reactions.

### Cell proliferation

For CFSE dilution, memory T cells were resuspended in PBS with 2% human serum and incubated with CFSE at the final concentration of 5 μg/ml for 8 minutes at 37°C, followed by quenching with complete medium and extensive washing prior to activation with plate-bound anti-CD3 and anti-CD28 antibodies. For BrdU incorporation, 100,000 primary T cells were plated overnight in 1 ml of complete medium in a 48-well plate. BrdU was incorporated at the concentration of 3 μg/ml for either 1 hour (day 3: stimulated primary T cells), 5.5 hours (day 5: stimulated cells), or 24 hours (day 6 or later time points after stimulation). After incorporation, cells were assayed using the APC BrdU Flow Kit by Pharmingen. For Jurkat cells, BrdU was incorporated for 5 hours.

### Western blotting

For protein extraction, T cells were washed with PBS and lysed in RIPA buffer (10 mM Tris-HCl pH 8.0, 1 mM EDTA, 1% Triton X-100, 0.1% sodium deoxycholate, 0.1% SDS, 140 mM NaCl) supplemented with a cocktail of protease inhibitors (P8340, Sigma, MO, USA.). Protein concentration was measured using a Pierce BCA assay (Thermo Fisher Scientific), and samples were either frozen or directly loaded onto 8% to 12% polyacrylamide gels. About 40 μg of total protein extract was used per sample. After electrophoresis, blotting on a PVDF membrane was performed using a wet transfer system and a methanol-based transfer buffer (20 mM Tris, 150 mM glycine, 20% methanol). Blocking was performed with 5% milk in TBST (5 mM Tris pH 7.3, 150 mM NaCl, 0.1% Tween-20) for 60 minutes at room temperature with gentle shaking. Blots were incubated with primary antibodies overnight at 4°C or 1 hour at room temperature, followed by washing and incubation with an HRP-conjugated secondary antibody. Blot development was performed using the ECL Prime Western Blotting Detection Reagent (Amersham, UK) and immediately analyzed with a blot imager (GE, Amersham Imager 680).

### Biotinylated miRNA:targets pull-down and sequencing

The biotinylated miRNA pull-down was performed as described [54] with optimization for primary human T helper cells. Briefly, 20 × 10^6^ memory T cells were stimulated with anti-CD3 and anti-CD28 antibodies and cultured for 5 to 6 days in complete medium. About 80 × 10^6^ cells were then transfected with 50 nM of biotinylated miRNA mimic or control oligonucleotide (Exiqon) using the 100 μl kit for NEON nucleofector (Thermo Fisher Scientific) in multiple transfections of 2.5 × 10^6^ cells each (2,200 V, 20 ms, 1 pulse). Immediately after transfection, cells were incubated in prewarmed complete medium without antibiotics supplemented with 60 U/mL of recombinant human IL-2. After 24 hours, cells were collected, washed with MACS buffer (PBS, 0.5% BSA, 2 mM EDTA), and lysed in 500 μl of Lysis Buffer (20 mM TRIS pH 8.0, NaCl 70 mM, KCl 150 mM, NP-40 0.5%, DTT 1 mM, glycerol 10%, EDTA 2 mM, RNAsin inhibitor (Promega) and protease inhibitor cocktail (Sigma)). Lysed cells were left 15 minutes on ice and then transferred at −80°C to ensure complete lysis. After thawing, the cell lysate was cleared of cell debris by centrifugation at 4°C for 20 minutes. A total of 30 μl of the cleared lysate were set aside and mixed with 90 μl of TRI-reagent for RNA extraction of the “input” fraction. The protein content in the remaining lysate was quantified with a BCA kit. Moreover, 100 μl of streptavidin agarose resin (Sigma) were washed twice with Lysis Buffer and subsequently incubated in Blocking Buffer (Lysis Buffer containing 1 mg/ml BSA, 100 μg/ml ssDNA Salmon Testis (Sigma), 500 μg/ml yeast tRNA (Thermo Fisher Scientific)) for at least 1 hour at 4°C on a wheel. All centrifugation steps with agarose resin were performed at 11,000 × *g* for 11 seconds. After 1 hour, the streptavidin agarose resin was washed 3 times with 500 μl of Lysis buffer and incubated overnight at 4°C on a spinning wheel with 1 mg of protein extract freshly supplemented with protein and RNAse inhibitors. The minimum volume of extract used for the incubation was 250 μl, at a final protein concentration of 1 to 3 mg/ml. The following day, samples were spun at 11,000 x *g* for 11 seconds. The agarose beads were washed 4 times with 1 ml of Lysis buffer, then incubated with 400 μl of TRI-reagent, 15 minutes at room temperature, and 1 hour 4°C. After centrifugation to remove the beads, the supernatants contained the “pull-down” fraction. Total RNA was extracted from the pull-down and input fractions using Zymo-Spin IC columns and quantified using a Qubit fluorometer. Sequencing was performed at the Next Generation Sequencing platform at the University of Bern (Switzerland), using an Illumina HiSeq 3000 (for miR-150, 1× 100-bp reads) or a NovaSeq 6000 (for miR-146a, 2× 50-bp reads). For library preparation, the Takara SMARTer Stranded Total RNA-Seq Kit v2—Pico Input Mammalian (Takara Bio, Japan) was used. FastQ files were analyzed using Linux and R. Quality control was performed with FastQC and FastQScreen. Reads were trimmed of overrepresented sequences with Cutadapt and mapped to the human GRCh37—hg19 assembly with Hisat2. Counts were generated using featureCounts and differential expression analysis performed with DESeq2.

### MiRNA seed analysis

miRWalk 2.0 [21] was used to predict miRNA targets. Predictions were done on 3′ UTR, CDS, and 5′ UTR. Afterward, the presence of at least a 6-mer was verified manually for each pull-down target.

### ATAC-seq library preparation

ATAC-seq was performed on 1 × 10^5^ sorted CD4^+^ memory and naive T cells obtained from 4 independent donors. After isolation, cells were either left resting or activated with plate-bound anti-CD3 and anti-CD28 antibodies in complete medium. Cells were processed on day 0 (resting condition), as well 1 and 3 days after activation. Briefly, cells were resuspended in 50 μl of lysis buffer (10 mM Tris-HCl pH 7.4, 10 mM MgCl_2_, 0.1% Igepal CA-630) and incubated on ice for 3 minutes followed by centrifugation (500 × *g*, 4°C, 20 minutes). Nuclei were then resuspended in 50 μl of tagmentation buffer (10 mM Tris-HCl, 25 mM MgCl_2_ and 1 μl of adaptor-loaded Tn5 transposase (produced in-house)) and incubated for 1 hour at 37°C. The cleanup of the tagmented DNA was performed by adding 10 μl of cleanup buffer (900 mM NaCl, 30 mM EDTA), 2 μl of 10% SDS, 6 μl of milliQ water, and 2 μl of Proteinase K (20 μg/μl) followed by incubation at 40°C for 30 minutes. Tagmented DNA was isolated using 2× AMPure XP beads and amplified by PCR with barcoded primers using 14 cycles of PCR. Finally, fragments smaller than 500 bp were purified with 0,65× AMPure XP beads and primers were removed by purification with 1,8× AMPure XP beads. Libraries were sequenced paired-end on an Illumina NextSeq500 platform.

### ATAC-seq analysis

Paired end reads were adapter trimmed using BBDuk in pair-end mode (https://jgi.doe.gov/data-and-tools/bbtools/bb-tools-user-guide/bbduk-guide, last modified version November 7, 2019). Reads were subsequently trimmed with trimmomatic in pair-end mode (version 0.39 flags: LEADING:3 TRAILING:3 SLIDINGWINDOW:4:15 MINLEN:25). Resulting reads were mapped to hg38 using bowtie2 (version 2.3.5.1; with flags:—very-sensitive -k 2 -t—phred33 -p 4 -q). Resulting bam files were then filtered using samtools (version 1.9) in order to remove unmapped reads, failing quality, mapping to mitochondrial chromosome, or with their mate unmapped (flag: samtools view -b -f 3 -F 524). Reads mapping to ENCODE black-list regions (https://github.com/Boyle-Lab/Blacklist) were also removed using bedtools pairToBed (version 2.29.2 flag: -type neither). PCR duplicated reads were removed using samtools markdup as described in the reference manual (http://www.htslib.org/doc/samtools-markdup.html). ATAC reads were then shifted as reported previously [70] using deepTools alignmentSieve (version 3.4.1; flag:—ATACshift). Peak calling was performed using MACS2 callpeak (version 2.2.6; options:–nomodel—format = BAMPE -B -g hs—call-summits). A reference set of peaks was created by selecting peaks called in each samples/replicate with qvalue ≤ 10^−10^ and being consistent between replicates (e.g., having an overlapping area of at least 50% between replicates). The resulting set of peaks was used to count reads in each sample using the R/Bioconductor package GenomicRanges and GenomicAlignment. Sample normalization was achieved by selecting invariant ATAC peaks across samples (for sample normalization strategy, see [71]). Differentially regulated peaks were selected using DESEq2 (R/Bioconductor package version 1.26.0; R version 3.6.2). Peak clustering was performed in R implementing a strategy similar to the one described by Dorrity and colleagues [72]. Transcription factor motif enrichment analysis was performed for each identified cluster using GimmeMotifs [44] using all accessible sites as background.

### CRISPR/Cas-9 gene editing and single-cell cloning

CRISPR**/**Cas**-**9 ribonucleoproteins (crRNPs) were delivered to primary CD4^+^ T memory cells by NEON transfection exactly as described [39]. Briefly, crRNAs and fluorescently labeled tracrRNAs (Dharmacon, IDT, NJ, USA) were mixed at a final concentration of 80 μM in 10 μl of Nuclease Free Duplex buffer (Dharmacon, IDT). The solution was then incubated for 5 minutes at 95°C and left at room temperature for 20 minutes for annealing. The ribonucleoprotein (RNP) complex was prepared immediately before transfection by mixing 7.5 μg of recombinant TrueCut Cas9 Protein v2 (Thermo Fisher Scientific) with 1.5 μl of the crRNA/tracrRNA duplex mix in a total volume of 3 μl followed by incubation for 20 minutes at room temperature. To increase transfection efficiency, Alt-R Electroporation enhancer (IDT) was added at a final concentration of 1.7 μM to the transfection mix (exclusively for primary cells). Transfection was performed with the 10 μl Neon Transfection System kit. One million memory T cells or Jurkat cells were resuspended in Buffer T or Buffer R, respectively, and added to the electroporation solution. Cells were then electroporated at 2,200 V, 20 ms, 1 pulse for primary cells and 1600 V, 10 ms, 3 pulses for Jurkat cells. After transfection, individual T cells were seeded in 384-well plates at 0.4 to 0.65 cells per well in complete medium in the presence of recombinant IL-2 (500 U/ml, produced in house), 1 μg/ml of phytohaemagglutinin (PHA), and 25,000 irradiated (45 Gy) allogeneic feeder cells (peripheral blood mononuclear cells, PBMCs) per well [39,73]. After 2 weeks, individual clones were transferred into round-bottom 96-well plates and further expanded for another 10 days in presence of IL-2, 500 U/mL.

### Analysis of CRISPR/Cas-9 deletion efficiency in T-cell clones

Genomic DNA (gDNA) from individual clones (approximately 1 × 10^5^ cells) derived from primary human T cells was isolated using the QIAamp DNA Micro Kit and DNeasy Blood & Tissue kit (Qiagen, Germany) or extracted with QuickExtract DNA extraction solution (Lucigen, WI, USA) following manufacturer’s protocol. To screen for the presence of deletions, a simple PCR spanning the region of interest was used. In the case of the *MIR150* gene, the 2 gRNAs were designed to produce a deletion of approximately 200 bp, and clones were screened for the presence of this deletion by PCR, using external primers. A similar strategy was used also for some *PDAP1*-deleted clones. To screen for the presence of mutations (rather than only large deletions) in *PDAP1* or *MYB*, a T7 endonuclease I cleavage assay was used [74,75]. Briefly, primers were designed to amplify an approximately 600- to 1,000-bp region surrounding the targeted area. PCR amplification was performed using the high fidelity KOD Hot Start DNA polymerase and 20 to 100 ng of gDNA template in 30 μl. Because the T7 endonuclease I cleaves mismatched heteroduplex DNA, 15 μl of each PCR product were denatured and reannealed to produce potential heteroduplexes of wild-type and mutated DNA strands. Five units of T7 endonuclease I (New England Biolabs, MA, USA) were added directly to the annealed PCR product and incubated at 37°C for 15 minutes. As control, parallel reactions without T7 endonuclease were performed. After resolution of the DNA bands on a 1% agarose gel, band intensities were quantified with ImageJ, and the percentage of cleavage efficiency was calculated by dividing the density of the cut product by that of the uncut. We considered as “modified” any clone presenting a cleavage efficiency higher than the average background generated by the control clones.

### Chromatin immunoprecipitation

About 60 million freshly isolated CD4^+^ T cells were resuspended in 36 ml of PBS and 1 mL of formaldehyde solution 37% (Sigma) to a final concentration of approximately 1%. After 10 minutes, the reaction was quenched with glycine at a final concentration of 0.125 M. After washing, cell pellets were flash-frozen in liquid nitrogen. Frozen cells were thawed and lysed in 3 mL of RIPA lysis buffer (Tris-HCl pH 8.0 10 mM, EDTA pH 8.0 1 mM, NaCl 140 mM, SDS 0.1%, deoxycholic acid 0.1%, phenylmethylsulfonyl fluoride 2 mM, 1× Sigma protease inhibitor) for 30 minutes on ice, followed by sonication using a Diagenode Bioruptor Plus, 4°C, 45 cycles 30 seconds on/60 seconds off, leading to DNA fragments of approximately 200 bp in size. At the end of the sonication process, chromatin was cleared by centrifugation and addition of 1% Triton X. Moreover, 20 μl of cleared chromatin were set aside as input. To assess the extent of shearing, part of the chromatin (100 μl) was de-crosslinked by incubation with 5 μg of proteinase K overnight at 65°C and column-purified before visual assessment on an agarose gel. For immunoprecipitation, 75 μl of magnetic Dynabeads protein G (Thermo Fisher Scientific) were washed twice in Binding Buffer (PBS, BSA 0.5%, Tween-20 0.5%) and incubated with 10 μg of mouse anti-human RFX5 antibody (sc-271756-X, Santa Cruz Biotechnology, TX, USA) in 300 μL of Binding Buffer for 2 hours at room temperature on a rotating platform. After washing, the beads were finally incubated with 1 ml of cleared chromatin overnight at 4°C on a rotating platform. After removal of the supernatant, the beads were washed 5 times with RIPA buffer, 2 times with Tris-HCl pH 8.0, 10 mM, EDTA pH 8.0 1 mM, NaCl 500 mM, SDS 0.1%, deoxycholic acid 0.1%, Triton X-100 1%, 2 times with LiCl buffer (Tris-HCl pH 8.0 10 mM, EDTA pH 8.0 1 mM, LiCl 250 mM, NP-40 0.5%, deoxycholic acid 0.5%), and once in Tris-EDTA buffer. Beads were then resuspended in 50 μL of Direct Elution Buffer (Tris-HCl pH 8.0 10 mM, EDTA pH 8.0 5 mM, NaCl 300 mM, SDS 0.5%) and treated with 5 μg of RNAse A, 37°C for 30 minutes. Glycogen 1 μl and 2.5 μl Proteinase K (20 mg/ml) were added, and samples were incubated at 37°C for additional 2 hours, shaking. Samples were finally reverse crosslinked by incubation at 65°C for 6 to 18 hours. Beads’ supernatants were transferred to new tubes and 132 μl of SPRI magnetic beads (Mag-Bind RxnPure Plus, Omega Bio-tek, GA, USA) were added to each sample and incubated 5 minutes at room temperature, followed by 2 washes with 500 μl of ethanol 70%, while leaving the tubes on the magnetic rack. After drying, the DNA was eluted with 30 to 60 μl of Tris-HCl pH 8.0, and DNA samples were quantified by Qubit fluorometric quantification (Invitrogen). Finally, 600 pg of immunoprecipitated DNA were used for qPCR, in a final volume of 10 μl.

### RNA immunoprecipitation

A total of 20 million memory T cells were isolated from healthy donors and activated with plate-bound anti-CD3 and anti-CD28 antibodies for 5 days. Cells from 2 distinct donors were then pooled together for a total of approximately 80 × 10^6^ cells. The cells were resuspended in ice-cold PBS at the density of 20 to 30 × 10^6^ cells/ml, and half of them were irradiated twice with 254 nm UV light at 0.2J using a UV Stratalinker. Each irradiation cycle was conducted on ice with 2-minute shaking intervals. After centrifugation, the cells were lysed in RIPA buffer (10 mM Tris-HCl, pH 8.0, 1 mM EDTA, 0.5 mM EGTA, 1% Triton X-100, 0.1% Sodium Deoxycholate, 0.1% SDS, 140 mM NaCl) with recombinant protease inhibitors (Sigma) and RNAse inhibitor (1 U/μl, Promega). Protein content was quantified using a Pierce BCA kit (Thermo Fisher Scientific), and 1 mg of cell extract was incubated on a rotating wheel approximately 16 hours at 4°C using 6 μg of antibody (anti-PDAP1 Bethyl (TX, USA) A304-651A or Proteintech (IL, USA) anti-Tubulin 66240-1-Ig). Upon addition of 60 μl of protein-G dynabeads (Thermo Fisher Scientific) and incubation for 4 hours at 4°C, beads were washed twice with RIPA buffer and 2 more times with RIPA-500 (same as RIPA, except for NaCl 500 mM). Washed beads were treated with proteinase K (60 mg in 60 μl of RIPA buffer containing RNAse inhibitor) at 37°C for 30 minutes, followed by RNA extraction using 400 μl of TRI-reagent (MRC) and Zymo spin RNA low-quantity IC columns. RNA was retrotranscribed using Quanta Bio master mix, and qPCR was performed using SYBR green reagents (Quanta Biosciences). For sequencing, total RNA was quantified using a Qubit fluorometer, followed by library preparation and sequencing at the Next Generation Sequencing platform of the University of Bern (Switzerland), using an Illumina NovaSeq 6000. For library preparation, the CORALL Total RNA-Seq Library Prep Kit was used. Quality control was performed with FastQC (v. 0.11.5) and RSeQC (v. 2.6.4). PCR duplicates were removed using UMI-Tools v1.1.1, and the resulting reads were mapped to the human GRCh38.104 assembly with Hisat2 (v. 2.1.0). Counts were generated using featureCounts (v. 1.6.0) and differential expression analysis performed with DESeq2. Only protein-coding transcripts were considered for further analysis.

### Poly-A RNA pull-down

mRNA pull-down was performed using the Dynabeads Oligo(dT)25 kit (Thermo Fisher Scientific) according to manufacturer’s instructions. Briefly, 60 to 80 × 10^6^ T cells were isolated and irradiated as described for RNA immunoprecipitation. Cells were then lysed directly in 1 ml of Lysis/Binding buffer and crude extracts were passed 20 times through a 21-gauge needle to decrease viscosity and then incubated directly with 100 μl of Dynabeads-oligo(dT) for 3 hours at 4°C on a rotating wheel. After extensive washing, beads were resuspended in 60 μl of Laemmli buffer (Tris-HCl pH 6.8 0.3125 M, glycerol 50%, β-mercaptoethanol 25%, sodium dodecyl sulfate 10%, bromophenol blue 0.1%) and boiled at 95°C for 5 minutes. Samples were then used for SDS-page and blotted with 2 μg/ml of anti-PDAP1 antibody.

### Immunofluorescence

Of note, 1 × 10^5^ memory T cells were fixed using 3.7% formaldeyde in PBS and then spun on cytospin slides (5 minutes at 500 rpm). The cells were then permeabilised with 0.1% Triton X in PBS, and blocking was performed using 10% goat serum in PBS. Slides were incubated with primary antibodies for 1 hour at room temperature, followed by washing and incubation with an secondary antibodies for 30 minutes at room temperature. Nuclei were counterstained with DAPI. Images were acquired with a Leica TCS SP5 laser scanning confocal microscope (LSCM), using a 63x/NA 1.4 PL APO CS Oil objective with a XY pixel size of 55 nm and pinhole 1 AU. Fluorescence was excited with 594 nm He:Ne laser and collected in range 600 to 700 nm. Quantification of the mean intensity of the signals in individual cells was performed using ImageJ software.

### Statistical analyses

Graphs were created with GraphPad Prism 8 software. FACS plots were analyzed by FlowJo 10 software. Heatmaps were made by R software using ggplot2, viridis, and reshape2 libraries. Statistical analyses were performed using GraphPad Prism 8. The comparison between 2 means was evaluated by parametric *t* test if the 2 populations compared were normally distributed or by nonparametric (Wilcoxon–Mann–Whitney) test in case the populations were not normally distributed. Distributions were tested using the Kolmogorov–Smirnov test. Welch correction *t* test was applied in case the 2 populations were heteroscedastic (distribution of populations was evaluated with an F-test). Comparisons among 3 or more sample means were made by ANOVA.

## Supporting information

S1 Data Excel spreadsheet containing, in separate sheets, the underlying numerical data for Figs 1A, 1B, 1C, 2A, 2B, 2C, 2D, 2E, 3A, 3B, 3C, 3D, 4A, 4C, 4D, 4E, 5B, 5C, 6A, 6B, 7B, 7C, and 7E and S1A, S1B, S1C, S1D, S2C, S3A, S3B, S5B, S5C, S6D, S7A, S7B, S7C, S9A, S9B, S9C, S10B, and S10C Figs. (XLSX)

S1 Fig miRNA expression in primary human T lymphocytes. **(A)** Naive, T_CM_, and T_EM_ lymphocytes were freshly separated from the peripheral blood of 4 independent donors. Moreover, 100 ng of total RNA were used for the analysis. Raw data were normalized to the top 25 most expressed miRNAs and considered as expressed if following thresholds applied: at least 1 sample with more than 125 normalized reads and no more than 1 sample containing less than 100 normalized reads. Mean ± SD. Each dot represents an independent donor. **(B)** Volcano plot representations of the same data an in (A), to compare miRNA expression across subsets. Differentially expressed miRNAs (Log_2_ ratio ≥ 1 and ≤ −1; Log_10_ *p*-value ≥ 1.3) are shown in red. *N* = 3 independent donors. **(C)** Naive, T_CM_ and T_EM_ cell subsets were isolated from peripheral blood and were either left resting or activated with plate-bound anti-CD3 and anti-CD28 antibodies for the indicated times. Total RNA was extracted and the expression of the indicated miRNAs measured by RT-qPCR. Data are shown as fold change compared to resting day 0 (d0) cells. *N* = 3 independent donors. **(D)** Jurkat T cells were transduced with a lentiviral vector (LV) to force miR-150 expression. Expression of miR-150 compared to control samples was measured by RT-qPCR (left), and cell proliferation was measured by BrdU incorporation assay (right). *N* = 4 independent experiments. Mean ± SD. Student *t* test, 2 tailed, paired. Underlying data can be found in S1 Data. FC, fold change; miRNA, microRNA; RT-qPCR, reverse transcription quantitative PCR.
(EPS)

S2 Fig Genomic deletion of 3 miR-150 binding sites in the PDAP1 3′ UTR abrogates miR-150 regulation. **(A)** Schematic representation of the *PDAP1* 3′ UTR with indicated the locations of the predicted miR-150 BS (black), the sgRNAs (red), and the primers used for gDNA screening (green arrows) of cells and clones lacking 3 or 1 single miR-150 site (ΔBS2/3/4 and ΔBS4, respectively). **(B)** Example of gDNA screening for single clones ΔBS4 (left) and ΔBS2/3/4 (right). We could identify only 2 clones with a partial deletion of ΔBS2/3/4. **(C)** Individual clones with the desired genomic modifications in the 3′ UTR of the *PDAP1* gene (or control clones, transfected with a scrambled sgRNA sequence) were pooled and transfected with either a miR-150 mimic or control oligonucleotide. Twenty-four hours after transfection, expression of *PDAP1* was measured by RT-qPCR (Scrambled control clones: *N* = 8 pooled clones, 2 experiments; ΔBS4: *N* = 9 pooled clones, 2 experiments; ΔBS2/3/4: *N* = 2 pooled clones). Underlying data can be found in S1 Data. BS, binding sites; gDNA, genomic DNA; PDAP1, PDGFA-associated protein 1; RT-qPCR, reverse transcription quantitative PCR; sgRNA, single-guide RNA; UTR, untranslated region.
(EPS)

S3 Fig miR-150 and MYB modulate T-cell proliferation. **(A)** Primary memory T cells and Jurkat cells were transfected with an miRNA mimic or transduced with a control or miR-150–expressing lentivirus. Seven days after transduction, *MYB* expression was measured by RT-qPCR. *N* = 3 (primary T) or *N* = 4 (Jurkat) independent experiments. Mean ± SD (primary T) or SEM (Jurkat). Student *t* test, 2 tailed, paired. **(B)** Memory T lymphocytes were loaded with CFSE, transfected with either siRNAs against *MYB* or a control oligonucleotide and activated with anti-CD3 and anti-CD28 antibodies. The extent of *MYB* down-regulation was measured by RT-qPCR (left), while cell proliferation was measured by CFSE dilution 4 days after activation (right). *N* = 3 independent experiments. Mean ± SD. Student *t* test, 2 tailed, paired. Underlying data can be found in S1 Data. CFSE, carboxyfluorescein succinimidyl ester; miRNA, microRNA; RT-qPCR, reverse transcription quantitative PCR; siRNA, small interfering RNA.
(EPS)

S4 Fig Poly-A mRNA pull-down recovers the PDAP1 protein. **(A)** Complete western blot images of the images shown in Fig 3A and **(B)** Fig 4B.
(EPS)

S5 Fig **(A)** RIP-seq for PDAP1 in memory T lymphocytes. Examples of snapshots of the sequencing tracks for selected genes. **(B)** Memory T lymphocytes were transfected with recombinant Cas-9 and sgRNAs against PDAP1 or scrambled controls. After cloning, expansion, and selection of *PDAP1*-deleted clones, the expression of the indicated mRNA transcripts was measured by RT-qPCR in individual clones. Each dot represents one clone (at least *N* = 8). Mann–Whitney test. Mean ± SD. **(C)** Memory T lymphocytes were transfected with siRNAs against *PDAP1* or *MYB*. After 24 hours, RNA was extracted, and the expression of *MYB* and *PDAP1* was measured by RT-qPCR. *N* = 3 independent donors. Mean ± SD. Student *t* test, 2 tailed, paired. Underlying data can be found in S1 Data. PDAP1, PDGFA-associated protein 1; RIP-seq, RNA immunoprecipitation sequencing; RT-qPCR, reverse transcription quantitative PCR; sgRNA, single-guide RNA; siRNA, small interfering RNA.
(EPS)

S6 Fig Screening strategies for CRISPR/Cas-9–targeted primary T-cell clones. **(A)** Clones targeted in the *MYB* gene were screened for deletion mutants by using PCR primers located externally to the gRNAs. **(B)** Clones targeted in the *PDAP1* gene were identified by PCR with or without T7 endonuclease I digestion. In the first screening strategy (left), a PCR product of 0.78 kb is detectable upon deletion of the genomic region between the 2 sgRNAs indicated in blue in the schematic representation, while the nondeleted band can be detected in some PCR reactions at approximately 5.4 kb. Shown are 9 deleted clones that were used for downstream analyses, one clone (H8) that showed no deletion and was discarded and one control clone. In a second screening strategy (right), when digested by T7 endonuclease I, a PCR product of approximately 600 bp is digested into 2 segments of 200 and 400 bp if a mutation or small indel is present in the amplified region. **(C)** Clones targeted in the *MIR150* gene were screened for the presence of a deletion across the miR-150 sequence by PCR, using primers located externally to the 2 gRNAs. In each case, the red dashed square highlights an example of deleted or mutated clone that was used for further analyses. **(D)** Jurkat T cells were transfected with Cas-9 RNPs to delete either *MYB* (left) or *PDAP1* (right). Cells were transfected with either 1 (blue line) or 2 (yellow line) gRNAs at the same time. Cell proliferation was measured 4 days after transfection by BrdU incorporation assay. gRNA, guide RNA; PDAP1, PDGFA-associated protein 1; RNP, ribonucleoprotein.
(EPS)

S7 Fig Deletion of the PDAP1 gene in B cell lymphoma cell lines led to reduced cell proliferation. **(A)** RT-qPCR and **(B)** western blot analyses for PDAP1 expression in the indicated cell lines and in freshly isolated primary human CD19^+^ B lymphocytes. **(C)** The *PDAP1* gene was deleted by CRISPR/Cas-9 in the indicated cell lines. After single-cell cloning, selection, and expansion, cell proliferation of the individual clones was measured by BrdU incorporation. Each dot represents one clone. Mean ± SD. Student *t* test, 2 tailed, unpaired. Underlying data can be found in S1 Data. PDAP1, PDGFA-associated protein 1; RT-qPCR, reverse transcription quantitative PCR.
(EPS)

S8 Fig ATAC-seq analysis in primary T cells. Clustering analysis including all ATAC-seq peaks that were significantly affected after 24 hours or 72 hours of activation in either naive or memory T cells. ATAC-seq, assay for transposase accessible chromatin and sequencing.
(EPS)

S9 Fig *RFX7* expression in human T lymphocytes. **(A)** Naive and memory T lymphocytes were stimulated with plate-bound anti-CD3 and anti-CD28 antibodies for 3 days. Total RNA was extracted and the expression of *RFX7* was measured by RT-qPCR. *N* = 2 independent donors (each dot represents one donor). Mean ± SD. This experimental setup, with 2 data points (average of technical duplicates for each donor), precludes a statistical assessment of the differences observed. **(B)** Naive T lymphocytes were stimulated with plate-bound anti-CD3 and anti-CD28 antibodies for the indicated times. Total RNA was extracted and the expression of the different *RFX* mRNAs was measured by RT-qPCR. *N* = 3 to 5 independent donors (each dot represents one donor). Mean ± SD. One-way ANOVA. **(C)** Resting primary memory CD4^+^ T lymphocytes were transfected with siRNAs against RFX5. Total RNA was extracted 24 hours after transfection, followed by RT-qPCR analyses of the indicated genes. Each dot represents one donor/experiment. Mean ± SD. Paired *t* test, 2 tailed. Underlying data can be found in S1 Data. RFX, regulatory factor X; RT-qPCR, reverse transcription quantitative PCR; siRNA, small interfering RNA.
(EPS)

S10 Fig Overexpression of RFX factors in activated cells is not sufficient to influence miR-150 expression and T-cell proliferation. **(A)** Resting memory T lymphocytes were transduced with the indicated plasmid (containing an IRES-GFP), followed by activation with plate-bound anti-CD3 and anti-CD28. Representative example of the efficiency of cell transduction. **(B)** GFP^+^ cells were sorted after 5 days, and proliferation was measured by BrdU incorporation assay. The results of *N* = 3 independent experiment is shown on the right. Each dot represents one donor. Mean ± SD. **(C)** A portion of the cells was used to measure the indicated transcripts by RT-qPCR. Each dot represents one donor. Mean ± SD. Underlying data can be found in S1 Data. RFX, regulatory factor X; RT-qPCR, reverse transcription quantitative PCR.
(EPS)

S1 Table NanoString profiling of miRNA expression in different primary human T-cell subsets. miRNA, microRNA.
(XLSX)

S2 Table Results of the pull-down and sequencing experiments for miR-150. (XLSX)

S3 Table Results of the pull-down and sequencing experiments for miR-146a. (XLSX)

S4 Table List of RBP genes identified at the intersection between the different experiments and cell lines shown in Fig 4C. RBP, RNA-binding protein.
(XLSX)

S5 Table RIP-seq results. RIP-seq, RNA immunoprecipitation sequencing.
(XLSX)

S6 Table ATAC-seq peaks. ATAC-seq, assay for transposase accessible chromatin and sequencing.
(XLSX)

S7 Table Motifs recovered from ATAC-seq analysis. ATAC-seq, assay for transposase accessible chromatin and sequencing.
(XLSX)

S8 Table Materials used in this study. (XLSX)

## Abbreviations

ATAC-seq: assay for transposase accessible chromatin and sequencing
BrdU: bromodeoxyuridine
CDS: coding sequence
CFSE: carboxyfluorescein succinimidyl ester
ChIP-seq: chromatin immunoprecipitation sequencing
crRNP: CRISPR/Cas-9 ribonucleoprotein
CTCL: cutaneous T-cell lymphoma
D^2^P^2^: Database of Disordered Protein Predictions
DICE: Database of Immune Cells
gDNA: genomic DNA
IRE1α: inositol-requiring enzyme 1α
LCSM: laser scanning confocal microscope
LNA: locked nucleic acid
MFI: mean fluorescence intensity
miRNA: microRNA
PBMC: peripheral blood mononuclear cell
PDAP1: PDGFA-associated protein 1
PEI: polyethylenimine
PHA: phytohaemagglutinin
PTCL: peripheral T-cell lymphoma
RBP: RNA-binding protein
RFX: regulatory factor X
RIP: RNA immunoprecipitation
RIP-seq: RNA immunoprecipitation sequencing
RNAi: RNA interference
RNP: ribonucleoprotein
RT-qPCR: reverse transcription quantitative PCR
sgRNA: single-guide RNA
siRNA: small interfering RNA
T_CM_: central memory T cells
TCR: T cell receptor
T_EM_: effector memory T cells
Treg: regulatory T cell
TSS: transcription start site
UTR: untranslated region
